# Next-generation sequencing of circulating tumor DNA to predict recurrence in triple-negative breast cancer patients with residual disease after neoadjuvant chemotherapy

**DOI:** 10.1038/s41523-017-0028-4

**Published:** 2017-07-03

**Authors:** Yu-Hsiang Chen, Bradley A. Hancock, Jeffrey P. Solzak, Dumitru Brinza, Charles Scafe, Kathy D. Miller, Milan Radovich

**Affiliations:** 1 Department of Medical & Molecular Genetics, Indiana University School of Medicine, Indianapolis, IN 46202 USA; 2 Department of Surgery, Indiana University School of Medicine, Indianapolis, IN 46202 USA; 3 Bioinformatics, Ion Torrent, Thermo Fisher Scientific, South San Francisco, CA 94080 USA; 4 Department of Medicine, Division of Hematology/Oncology, Indiana University School of Medicine, Indianapolis, IN 46202 USA; 5 Indiana University Melvin and Bren Simon Cancer Center, Indiana University School of Medicine, Indianapolis, IN 46202 USA; 6 Indiana University Center for Computational Biology and Bioinformatics, Indiana University School of Medicine, Indianapolis, IN 46202 USA

## Abstract

Next-generation sequencing to detect circulating tumor DNA is a minimally invasive method for tumor genotyping and monitoring therapeutic response. The majority of studies have focused on detecting circulating tumor DNA from patients with metastatic disease. Herein, we tested whether circulating tumor DNA could be used as a biomarker to predict relapse in triple-negative breast cancer patients with residual disease after neoadjuvant chemotherapy. In this study, we analyzed samples from 38 early-stage triple-negative breast cancer patients with matched tumor, blood, and plasma. Extracted DNA underwent library preparation and amplification using the Oncomine Research Panel consisting of 134 cancer genes, followed by high-coverage sequencing and bioinformatics. We detected high-quality somatic mutations from primary tumors in 33 of 38 patients. TP53 mutations were the most prevalent (82%) followed by PIK3CA (16%). Of the 33 patients who had a mutation identified in their primary tumor, we were able to detect circulating tumor DNA mutations in the plasma of four patients (three TP53 mutations, one AKT1 mutation, one CDKN2A mutation). All four patients had recurrence of their disease (100% specificity), but sensitivity was limited to detecting only 4 of 13 patients who clinically relapsed (31% sensitivity). Notably, all four patients had a rapid recurrence (0.3, 4.0, 5.3, and 8.9 months). Patients with detectable circulating tumor DNA had an inferior disease free survival (*p* < 0.0001; median disease-free survival: 4.6 mos. vs. not reached; hazard ratio = 12.6, 95% confidence interval: 3.06–52.2). Our study shows that next-generation circulating tumor DNA sequencing of triple-negative breast cancer patients with residual disease after neoadjuvant chemotherapy can predict recurrence with high specificity, but moderate sensitivity. For those patients where circulating tumor DNA is detected, recurrence is rapid.

## Diagnostics: Liquid biopsy detects recurrence but lacks sensitivity

A blood test can detect disease recurrence among women with triple-negative breast cancer (TNBC), but lacks sensitivity for distant relapse. A team led by Milan Radovich from Indiana University School of Medicine in Indianapolis, USA, analyzed tumor and blood plasma samples from 38 patients with TNBC who underwent neoadjuvant chemotherapy to see whether they could find evidence of mutated tumor DNA circulating in the blood after surgery that might predict cancer relapse. This kind of “liquid biopsy” has shown promise for patients with metastatic disease, but it has been tested less extensively in people with earlier-stage disease. The researchers’ blood test picked up tumor DNA in four patients after surgery, all of whom experienced disease recurrence. However, it missed nine other cases of relapse—indicating that the test has high specificity, but only moderate sensitivity.

## Introduction

Triple-negative breast cancer (TNBC) is defined by the absence of estrogen-receptor (ER), progesterone-receptor (PR), and human epidermal growth factor receptor 2 (HER2) over-expression.^1–4^ While TNBC comprises a minority of breast cancer cases (15–20%), it results in a disproportionally higher rate of mortality. Compared to ER- and HER2-postive disease, TNBCs have a higher incidence of visceral metastasis, a higher likelihood of relapse within the first 3 years after chemotherapy and surgery, and a shorter overall survival (OS) after the onset of metastatic disease.^5,6^

A significant proportion of patients with TNBC are treated with neoadjuvant chemotherapy. A stark dichotomy exists in outcome based on response to neoadjuvant therapy. Approximately, a third of patients will achieve a pathologic complete response (pCR), and go on to have a favorable OS outcome (94% at 3 years). In contradistinction, two-thirds of patients have residual disease after neoadjuvant chemotherapy and are at a high risk of relapse leading to an inferior OS (68% at 3 years).^7^ Methods that can detect the presence of tumor material in the circulation of patients who are technically “disease-free” after neoadjuvant chemotherapy and surgery may be used to predict those patients whose disease will recur, and further help to determine, which patients may need additional therapy.

An emerging method for non-invasive cancer detection is the analysis of circulating tumor DNA (ctDNA), also known as “liquid biopsies”. ctDNA is released into the circulation from the apoptosis or necrosis of tumor tissue, or from circulating tumor cells (CTCs) present in blood.^8,9^ It has been demonstrated that ctDNA can be detected in many types of cancer, including: breast,^10–14^ prostate,^15^ gastric,^16^ and others.^8^ The fraction of ctDNA compared to total cell-free DNA, can be quite small, in many cases <1%.^17–19^ Highly sensitive next-generation sequencing techniques though, can be used to detect low amounts of ctDNA. Herein, using plasma samples from a completed Phase II clinical trial of TNBC patients with residual disease after neoadjuvant chemotherapy, we applied next-generation DNA sequencing to determine if detection of ctDNA can be used as a predictor of relapse in this high-risk patient population.

## Results

### Patient and sample selection

**Fig. 1:**
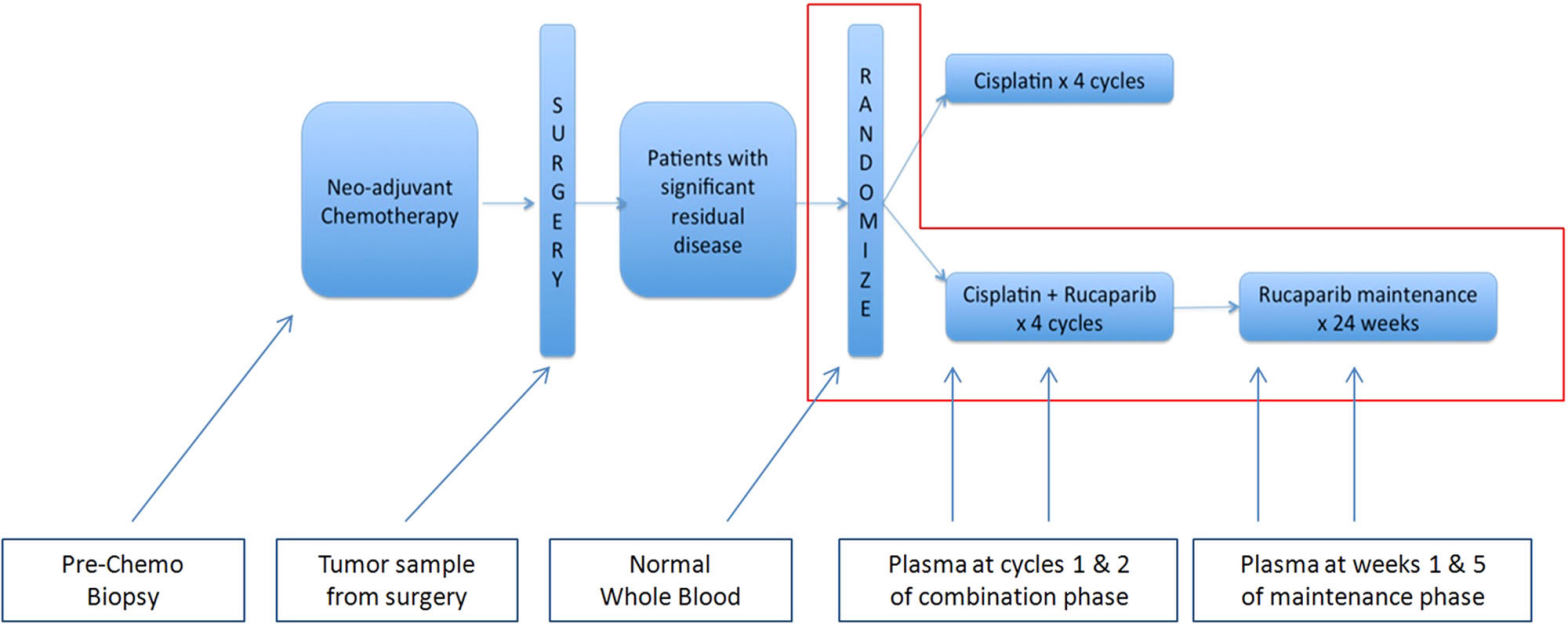
Trial schema for BRE09-146.BRE09-146 was a Phase II clinical trial to evaluate 2-year disease-free survival (DFS) in TNBC patients, treated with either Cisplatin (Arm A) or Cisplatin in combination with PARP inhibitor Rucaparib (Arm B) after neoadjuvant chemotherapy. Tumor tissue, whole blood, and plasma from four time points after surgery were collected as indicated. In this trial, plasma samples were collected only in Arm B of (the area enclosed by the *red rectangle*). Plasma samples were collected at four timepoints: Cycles 1 and 2 of the combination phase, and during weeks 1 and 5 of the maintenance phase

**Fig. 2:**
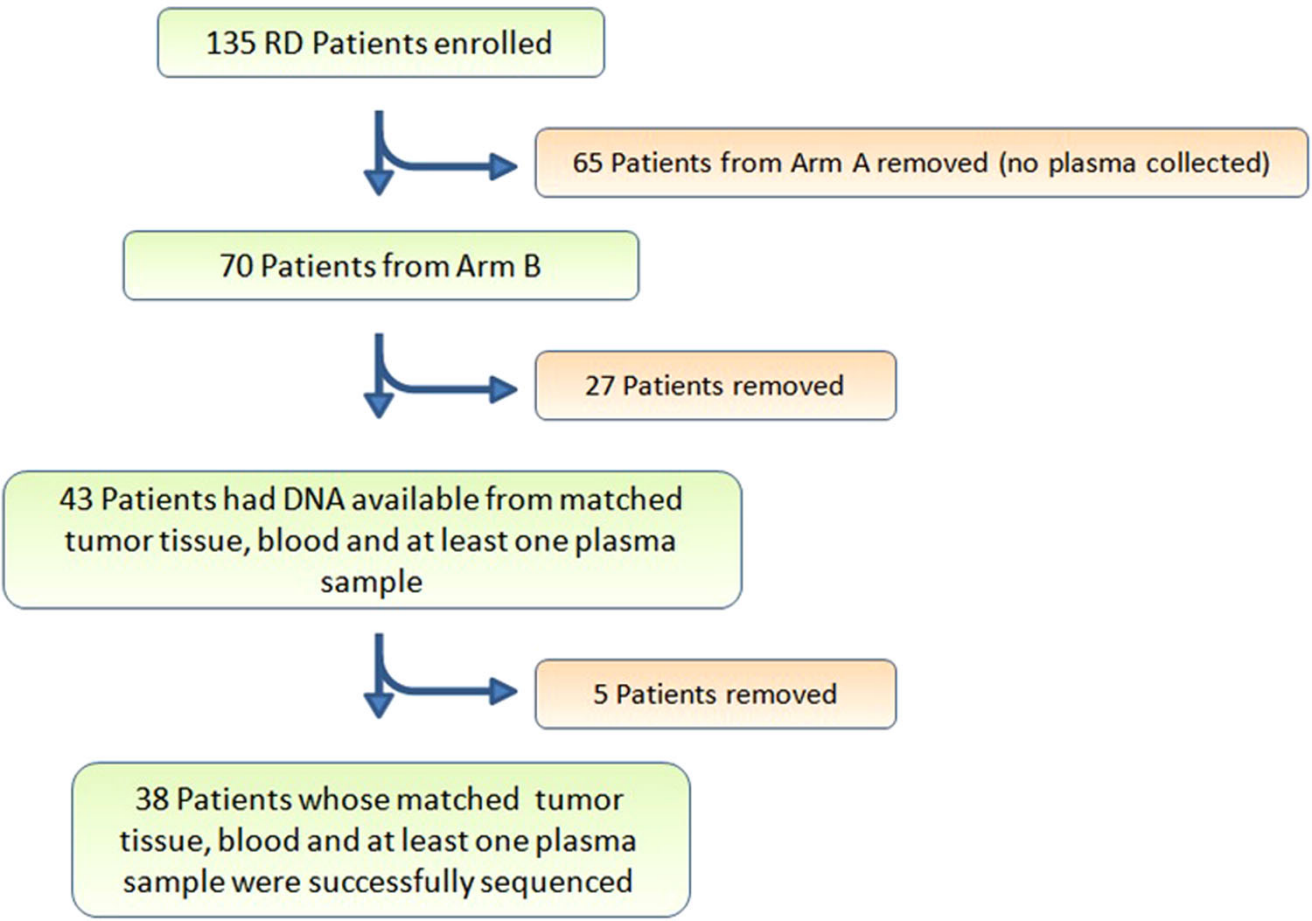
CONSORT diagram. There were 135 patients enrolled in BRE09-146. In this study, we focused on 70 patients from Arm B. In Arm B, 27 patients did not have matched tumor tissue, whole blood, and at least one plasma collection and were excluded from this study leaving an *N* = 43. A further five patients were removed due to the inability to successfully create a plasma DNA library. In total, 38 patients reached the criteria for analysis

One-hundred thirty-five patients were enrolled on the BRE09-146 clinical trial. Patient characteristics, including: median age, race, neoadjuvant chemotherapy, radiation therapy, median residual lymph node positivity (LN+), and median residual cancer burden (RCB) are detailed in Table 1. All patients received multiple agent neoadjuvant chemotherapy, with the vast majority receiving a combination of anthracycline, cyclophosphamide, and paclitaxel, followed by surgery and radiotherapy (Table 1). TNBC patients who completed neoadjuvant therapy and had significant residual disease were randomized to either cisplatin monotherapy or the combination of cisplastin plus rucaparib (Fig. 1) (See methods for details). Plasma samples used for the analysis of ctDNA were only collected in patients enrolled in the combination arm (Fig. 1). Details of patient selection included in this study are outlined in the Consolidated Standards for Reporting Trials (CONSORT) diagram (Fig. 2). In total, 38 patients with matched tumor tissue, blood, and at least one plasma sample were successfully sequenced (Fig. 2).

**Table 1:** Clinical characteristics of patients enrolled on the BRE09-146 clinical trial

BRE09-146	Arm A cisplatin (*n* = 65)	Arm B cisplatin + rucaparib (*n* = 70)	Subjects from arm B for this study (*n* = 38)
Median age	48 (27–69)	47 (21–75)	47 (21–66)
Race			
a. African American	20.0%	18.6%	13.2%
b. White	75.4%	72.9%	73.7%
c. Asian	1.5%	4.3%	7.9%
d. Others	3.1%	4.3%	5.3%
Neoadjuvant chemotherapy			
a. Anthracycline	89.2%	88.6%	92.1%
b. Cyclophosphamide	95.4%	90.0%	92.1%
c. Taxane	95.4%	92.9%	92.1%
d. Carboplatin	1.5%	10.0%	10.5%
e. Unknown	3.1%	2.9%	0%
Radiation therapy	87.7%	85.7%	84.2%
Median residual lymph node positivity (LN+)	1 (0–15)	1 (0–38)	1 (0–38)
Median residual cancer burden	2.6 (0–5.0)	2.7 (0–5.3)	3.1 (0–5.3)

### Identification of somatic mutations in the primary tumors

**Fig. 3:**
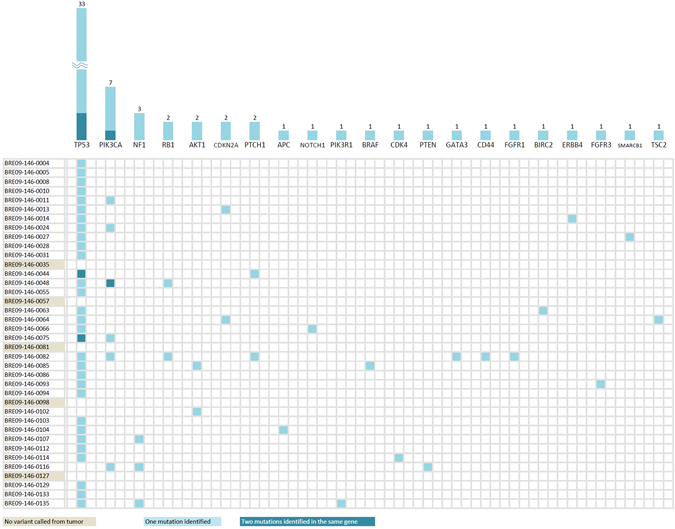
Somatic mutations identified from sequencing of tumor tissues. Among the 38 patients in our study, 33 of them had at least one somatic mutation identified (87%); 21 of them had two or more somatic mutations (55%). TP53 mutations were the most prevalent in this study, followed by PIK3CA pathway mutations. Notably, there were 14 different mutations exclusively present in individual patients, representing the genomic heterogeneity of TNBC patients

We first identified somatic mutations present in the primary tumor by identifying variants from tumor sequencing that were not present in the matched normal blood. Of the 38 patients described above, we successfully identified at least one somatic mutation in 87% of patients (33 of 38; Fig. 3), and two or more somatic mutations in 55% of patients (21 of 38; Fig. 3). Among those who had somatic mutation(s) identified, 31 patients had TP53 mutations (33 TP53 mutations in total; two patients had dual TP53 mutations). Ten out of 38 patients carried genetic alterations in the genes involved in PI3K signaling pathway. Among those, PIK3CA was the most common gene with genetic alterations (six patients), followed by AKT1 (two patients), PIK3R1, and PTEN (one patient each). The high-rate of TP53 and PI3K mutations is congruent with published data from the The Cancer Genome Atlas (TCGA),^20^ which observed the same pattern in TNBC tumors.

### Detection of somatic mutations (ctDNA) in matched plasma samples

**Fig. 4:**
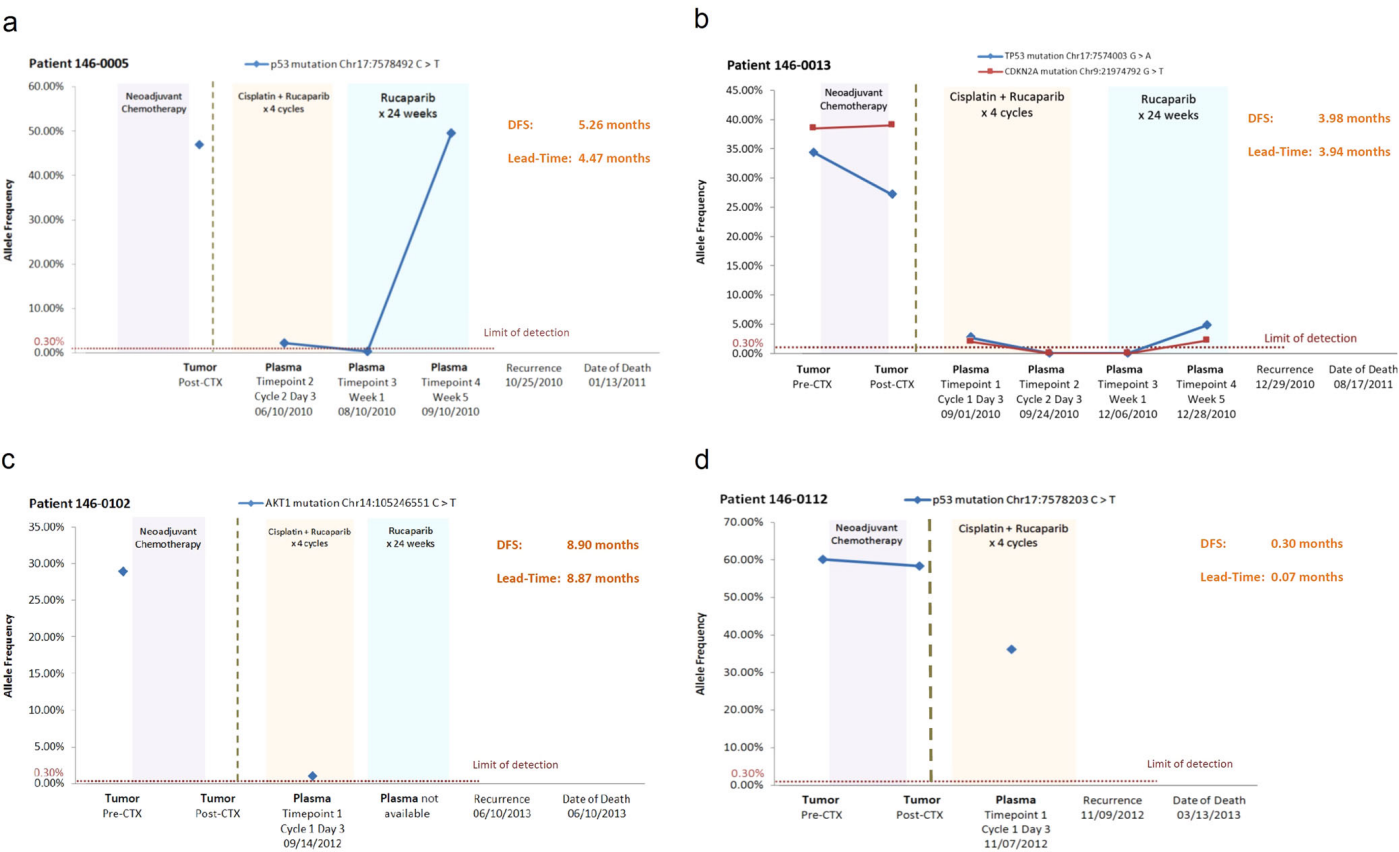
Longitudinal allele frequency tracking of ctDNA mutations. Somatic mutations were first identified in the primary tumor. These mutations were then searched for in matched plasma samples. ctDNA mutations were identified in four patients at varying allele frequencies. From patient 146-0005(a) and patient 146-0013(b), the increasing allele frequency of ctDNA was observed before clinically recurrence was diagnosed. Patient 146-0102(c) and patient 146-0112(d) had only one timepoint plasma sample available, and we were able to detect the ctDNA before clinical recurrence as well. The lead-time range was 0.07 to 8.87 months

**Fig. 5:**
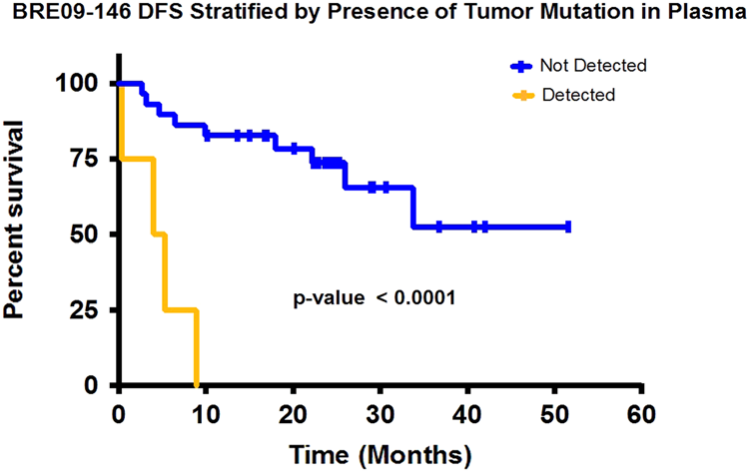
Kaplan–Meier plot: disease-free survival stratified by presence of tumor mutation in plasma. Four patients from this study who had mutation identified from plasma samples relapsed rapidly (0.3, 4.0, 5.3, and 8.9 months). The *yellow line* represents patients with detectable ctDNA in plasma. The *blue line* represents patients with no detectable ctDNA in plasma.The difference in median DFS between patients with detectable ctDNA vs. those without was statistically significant (*p* < 0.0001, median DFS: 4.6 mos. vs. NR; HR = 12.6, 95% CI: 3.06-52.2)

We then searched for the somatic mutations identified from the primary tumors in the matched plasma samples. Of the 33 patients who had a somatic mutation identified in their primary tumor, we were able to detect somatic mutations in the plasma of four patients (three TP53 mutations, one AKT1 mutation, and one CDKN2A mutation). All four patients had recurrence of their disease (100% specificity), but sensitivity was limited to detecting only 4 of 13 patients who relapsed (31% sensitivity). Figure 4 details the time-course of the mutational allele frequency for these four patients. In patient 146-0005 (Fig. 4a), a TP53 mutation (Chr17:7578492, C to T) was detected in timepoint 2 and 4 plasma samples. A similar pattern (timepoint 1 and 4) was observed in patient 146-0013 who had a different TP53 mutation (Chr17:7574003, G to A) and a CDKN2A mutation (Chr9:21974792) (Fig. 4b). We were also able to detect somatic mutations in plasma samples from the other two patients who had only one timepoint plasma sample available (146-0102, AKT1 mutation, Chr14:105246551, C to T; 146-0112, TP53 mutation, Chr17:7578203, C to T) (Fig. 4c, d). All mutations were located in exonic regions. Interestingly, all four patients had a rapid recurrence: average of 4.6 months (0.3, 4.0, 5.3, and 8.9 months; Fig. 4). The lead time of detection of the mutation in the plasma to clinical recurrence ranged from 0.07 to 8.87 months (Fig. 4). A Kaplan–Meier plot demonstrates that the patients who had ctDNA detected in their plasma, had a significantly inferior disease-free survival (DFS) compared to patients where ctDNA was not detected (*p* < 0.0001, median DFS: 4.6 mos. vs. not reached (NR); hazard ratio (HR) = 12.6, 95% confidence interval (CI): 3.06–52.2) (Fig. 5). In multivariate Cox regression analysis, with the addition of RCB and number of positive lymph nodes as covariates, the detection of ctDNA remained independently associated with inferior DFS (*p* = 0.011, median DFS: 4.6 mos. vs. NR; HR = 8.6, 95% CI: 1.6–45.7).

We were also interested in whether subclonal mutational evolution occurred after chemotherapy, generating new mutations (not present in the primary tumor) that could be detected in the plasma. To examine this, we analyzed the sequencing data for mutations which were exclusively present in the plasma samples from all 38 patients, no matter who had disease recurrence or not. This analysis did not identify any de novo mutations exclusively in the plasma, suggesting that only mutations first identified in the primary tumor were detectable in the plasma.

## Discussion

TNBC patients who do not achieve a pCR to neoadjuvant chemotherapy are at a high-risk of recurrence from their disease. Unfortunately, there is no FDA approved standard-of-care for this post-neoadjuvant setting. However, reported results from the CREATE-X trial (presented at 2015 San Antonio Breast Cancer Symposium) demonstrated an improvementin 2-year DFS and OS with the use of post-neoadjuvant Capecitabine for women with HER2-negative breast cancer with residual disease after neoadjuvant chemotherapy.^21^ A subgroup analysis revealed an improved benefit for TNBC patients.^21^ Given the clinical scenario, determining those patients who will relapse using methods that can detect the presence of tumor material, even when the patient is technically “disease-free” after surgery, can help predict which patients will recur, and potentially design therapeutic strategies for this population. Although tissue biopsy remains the standard approach for determining the presence of tumor, the so-called “liquid biopsy” using ctDNA is emerging as a complimentary method. Because somatic mutations provide intrinsic specificity for nucleic acid material derived from tumor tissue, the presence of ctDNA implies the presence of disease. In the evolving realm of circulating biomarkers, a recent study suggests that ctDNA may confer the highest sensitivity. Dawson et al.^13^ compared the use of circulating antigen 15-3, CTCs and ctDNA for blood-based detection, and demonstrated that the measurement of ctDNA possessed the highest sensitivity for monitoring metastatic breast cancer. While the vast majority of ctDNA studies have focused on patients with metastatic disease, in this study we focused on patients who are in the curative setting. The patients in our cohort are disease free by standard clinical assessment, but are known to be at a high-risk of relapse.

In our study, we searched for somatic mutations in plasma-sequencing data that were first identified in the matched primary tumor. Congruent with published studies of genomic sequencing of TNBCs, we observed a high-rate of TP53 and PI3K pathway mutations.^20^ Of 33 evaluable patients, 13 had a clinical relapse, and of those, we were able to detect ctDNA in 4. Of interest, all four of these patients had a rapid recurrence, ranging from 0.3 months—8.9 months. Our lead-time from the first-detection of ctDNA to clinical recurrence ranged from 0.07 months—8.87 months. We were unable to detect ctDNA in patients with distant recurrence. Further, we were unable to detect ctDNA in five patients who had a recurrence in <12 months. While all patients in which ctDNA was observed did have a rapid recurrence, the low sensitivity to detect distant, and in some cases rapid recurrence, highlights its limitations. Because the ability to detect ctDNA is proportional to the number of mutated molecules in the circulation; disease burden, and the volume of plasma that is sampled are important factors that regulate sensitivity. Our study represents a “worst-case scenario” in which there is no detectable disease burden at enrollment, and only 1 mL of plasma in which to perform our studies. Even in this setting, we were able to detect some patients with rapid recurrence. Given the retrospective nature of our study with a limited sample size, however, a prospective trial to prove clinical utility is well warranted.

A pivotal study by Garcia-Murillas et al.^14^ in a cohort of early breast cancer patients demonstrated that detection of ctDNA showed a similar pattern of rapid recurrence. A similar study by Olsson et al. showed that serial ctDNA sampling in patients with primary breast cancer can reach an average lead time of 11 months before the occurrence of metastatic disease.^22^ Key differences between our studies and theirs is the specific enrichment of a TNBC population in our study, and our use of next-generation sequencing vs. digital-droplet PCR (ddPCR) for ctDNA detection. While ddPCR has increased sensitivity, it requires the generation of patient-specific custom assays, thus next-generation sequencing has the advantage of being more generalizable for the application of ctDNA detection to a breast cancer population. Lastly, another study by Riva et al.,^23^ the investigators were unable to detect ctDNA in TNBC patients after surgery using ddPCR. This observation along with ours highlights the importance of serial sampling after surgery.

In conclusion, next-generation ctDNA-sequencing of TNBC patients after neoadjuvant chemotherapy and surgery can detect rapid-recurrence but sensitivity to detect distant recurrence is limited. Further studies that can capitalize on strategies to increase sensitivity are well warranted. Indeed, novel extraction methodologies; sequencing chemistries that attempt to provide increased enrichment of mutated DNA molecules (i.e., CAPP-Seq^24^); sensitive nucleic acid detection using CRISPR-Cas13a^25^ along with isothermal amplification; or the combination of ctDNA with other blood-based biomarkers such as miRNA, lncRNA, or exosomal protein;^26^ are all further avenues of exploration.

## Patients and methods

### Clinical trial and correlative samples

BRE09-146 was a prospective, multi-site, Phase II clinical trial of Cisplatin + PARP inhibition in TNBC patients who have residual disease after neoadjuvant chemotherapy. Eligibility criteria required residual disease, defined as either: (i) residual tumor >2 cm in the breast; (ii) lymph node involvement; or (iii) RCB classification of II or III. Eligible patients were then randomized either to Cisplatin for four cycles or Cisplatin plus the PARP inhibitor Rucaparib for four cycles followed by maintenance Rucaparib for 24 weeks (Fig. 1). Patient were enrolled on trial from March 2010 to May 2013. BRE09-146 is registered on ClinicalTrials.gov (https://clinicaltrials.gov/ct2/show/study/NCT01074970). For correlative analyses, clinical sites submitted tumor biopsies from the time of diagnosis, tumor from residual disease at the time of surgery, as well as whole blood prior to treatment. From the combination arm only (Cisplatin + Rucaparib), plasma samples that were originally collected for pharmacokinetic analyses, were obtained at four pre-defined timepoints: Cycles 1 and 2 of the combination phase, and during Weeks 1 and 5 of the maintenance phase (detailed in Fig. 1). For this correlative study of ctDNA, each evaluable patient had to have at least one tumor sample (with 60% or greater tumor cellularity), one whole blood sample, and one plasma sample submitted. Tumor DNA was isolated from formalin-fixed paraffin embedded (FFPE) tissue using the Qiagen AllPrep DNA/RNA FFPE kit. Whole blood was isolated using AutogenFlex Star instrument and the Flexigene AGF3000 blood kit at the Indiana Clinical and Translational Sciences Institute Specimen Storage Facility (ICTSI-SSF). Plasma DNA was isolated from 1 ml of plasma using the Qiagen QIAamp Circulating Nucleic Acid Kit. All DNA samples were quantified using the Qubit dsDNA HS Assay Kit (Life Technologies). The trial and correlative studies were approved by the Indiana University Institutional Review Board (IRB); patients provided written informed consent prior to study entry including consent for blood samples for genomic analysis. The study was conducted in accordance with appropriate protocols established by Indiana University.

### Library preparation and sequencing

DNA samples from each tumor, blood, and plasma sample underwent the same procedure for library preparation. DNA samples were amplified using a highly-multiplexed polymerase chain reaction (PCR) that amplifies 134 genes that are well-known to be mutated in cancer (Ion Ampliseq Oncomine Research Panel) (see Supplementary Methods). Libraries were completed using the Ion Ampliseq Library Kit (see Supplementary Methods). The libraries were subjected to emulsion PCR, and prepared for sequencing using the Life Technologies Ion Chef and the Ion PI IC 200 Kit (Life Technologies). Up to seven different barcoded libraries were loaded onto one Ion PI v2 BC chip to obtain appropriate coverage. Sequencing was carried out using a Life Technologies Ion Proton Next-generation sequencer (Supplementary Fig. 1). Each sample in our study was sequenced to at least 2500× coverage, with a median coverage of 6071× (range 2559×–13995×). Coverage details and allele frequencies of all observed mutations are detailed in Supplementary Table 1.

### Bioinformatics analysis

Each sequencing run produced approximately 56–89 million reads. Reads underwent primary analysis using the Torrent Suite v4.2.1, which includes quality control, read trimming, and mapping to the human genome (hg19). Variant calling was performed using the Torrent Variant Caller v4.2.1.0. Somatic mutations were identified by comparing variants observed in the tumor sample that were not present in a matched blood sample. Identified somatic variants were then searched for in the plasma DNA sequencing using the Torrent Variant Caller. We also manually inspected called variants using the integrative genomics viewer^27, 28^ to confirm the presence of variants and to rule out false positives.

### Statistical analysis

Clinical follow-up data was provided by the trial contract research organization: Hoosier Cancer Research Network. The median follow-up for DFS for the entire trial was 24 months. DFS analysis was performed using Cox regression (IBM SPSS Statistics version 24) and plotted using the Kaplan–Meier method (Graphpad Prism, GraphPad Software, Inc.). In univariate Cox regression analysis, the detection of ctDNA was significantly associated with inferior DFS (see Results). In multivariate Cox regression, when adding RCB and number of positive lymph nodes as covariates, the detection of ctDNA was independently associated with DFS (see Results). Age, race, tumor grade, and Eastern Cooperative Oncology Group (ECOG) performance status were not associated with DFS, and not used as covariates.

### Data availability

The detailed sequencing results of coverage numbers, allele frequency, and tumor cellularity are available in Supplementary Table 1.

## Electronic supplementary material

Supplementary Method

Experimental workflow of mutation identification

Supplementary Table
