# Supplementary material for: Next-generation sequencing of circulating tumor DNA to predict recurrence in triple-negative breast cancer patients with residual disease after neoadjuvant chemotherapy
Source: NPJ Breast Cancer. 2017 Jul 3;3:24. doi: 10.1038/s41523-017-0028-4 (PMC5495776; doi:10.1038/s41523-017-0028-4)
Supplement: Supplementary file 1 — Supplementary Method [file 41523_2017_28_MOESM1_ESM.docx]

**SUPPLEMENTARY METHODS**

**Ion Ampliseq Oncomine Research Panel**

For this study, we used the Ion Ampliseq Oncomine Research Panel that amplifies 134 genes that are commonly mutated in cancer. Only the DNA portion of the panel was used as follows:

Hotspot genes (n=66):

| ABL1 | CTNNB1 | FOXL2 | JAK2 | MPL | RAF1 |
| --- | --- | --- | --- | --- | --- |
| AKT1 | DNMT3A | GATA2 | JAK3 | MTOR | RET |
| ALK | EGFR | GNA11 | KDR | MYD88 | RHEB |
| AR | ERBB2 | GNAQ | KIT | NFE2L2 | RHOA |
| BRAF | ERBB3 | GNAS | KRAS | NRAS | SF3B1 |
| BTK | ERBB4 | HRAS | MAGOH | PAX5 | SMO |
| C15orf23 | ESR1 | IDH1 | MAP2K1 | PDGFRA | SPOP |
| CBL | EZH2 | IDH2 | MAPK1 | PIK3CA | SRC |
| CDK4 | FGFR2 | IFITM1 | MAX | PPP2R1A | STAT3 |
| CHEK2 | FGFR3 | IFITM3 | MED12 | PTPN11 | U2AF1 |
| CSF1R | FLT3 | JAK1 | MET | RAC1 | XPO1 |

Copy number variants (n=43):

| ACVRL1 | CCND1 | ERBB2 | KIT | MYCN | TERT |
| --- | --- | --- | --- | --- | --- |
| AKT1 | CCNE1 | FGFR1 | KRAS | NKX2-1 | TIAF1 |
| AR | CD274 | FGFR2 | MCL1 | PDGFRA | ZNF217 |
| APEX1 | CD44 | FGFR3 | MDM2 | PIK3CA |  |
| BCL2L1 | CDK4 | FLT3 | MDM4 | PNP |  |
| BCL9 | CDK6 | GAS6 | MET | PPARG |  |
| BIRC2 | CSNK2A1 | IGF1R | MYC | RPS6KB1 |  |
| BIRC3 | EGFR | IL6 | MYCL1 | SOX2 |  |

Tumor suppressors (n=25):

| APC | CDH1 | NF1 | PTEN | TP53 |
| --- | --- | --- | --- | --- |
| ATM | CDKN2A | NF2 | RB1 | TSC1 |
| BAP1 | FBXW7 | NOTCH1 | SMARCB1 | TSC2 |
| BRCA1 | GATA3 | PIK3R1 | SKT11 | VHL |
| BRCA2 | MSH2 | PTCH1 | TET2 | WT1 |

**Library Preparation**

The PCR program for amplification using the Ion Ampliseq Oncomine Research Panel was as follows: 1 x (99°C for 2 minutes), 21 x (99°C for 15 seconds, 60°C for 4 minutes), hold at 10°C. After amplifying DNA targets, DNA amplicons of the same sample were combined in one well of a 96-well plate. The two ends of the amplicons were then partially digested by FuPa Reagent (50°C for 10 minutes, 55°C for 10 minutes, 60°C for 20 minutes, hold at 10°C up to 1 hour), followed by barcoded adapter ligation (22°C for 30 minutes, 72°C at 10 minutes, hold at 10°C up to 1 hour). The libraries were then purified by AMPure XP reagent at room temperature.

The concentration of the eluted library was quantified by qPCR with the Ion Library Quantitation Kit (Cat. No. 4468802) using the Life Technologies 7900HT Fast Real-Time PCR System. The PCR program was as follows: 1 x (95°C for 20 seconds), 40 x (95°C for 1 second, 60°C for 20 seconds). A library concentration of 50 picomolar or greater qualified for subsequent steps of Ion Chem emulsion PCR and templating and sequencing.
