# Supplementary material for: Next-generation sequencing of circulating tumor DNA to predict recurrence in triple-negative breast cancer patients with residual disease after neoadjuvant chemotherapy
Source: NPJ Breast Cancer. 2017 Jul 3;3:24. doi: 10.1038/s41523-017-0028-4 (PMC5495776; doi:10.1038/s41523-017-0028-4)
Supplement: Supplementary file 2 — Experimental workflow of mutation identification [file 41523_2017_28_MOESM2_ESM.docx]

**Supplementary Figures**

**
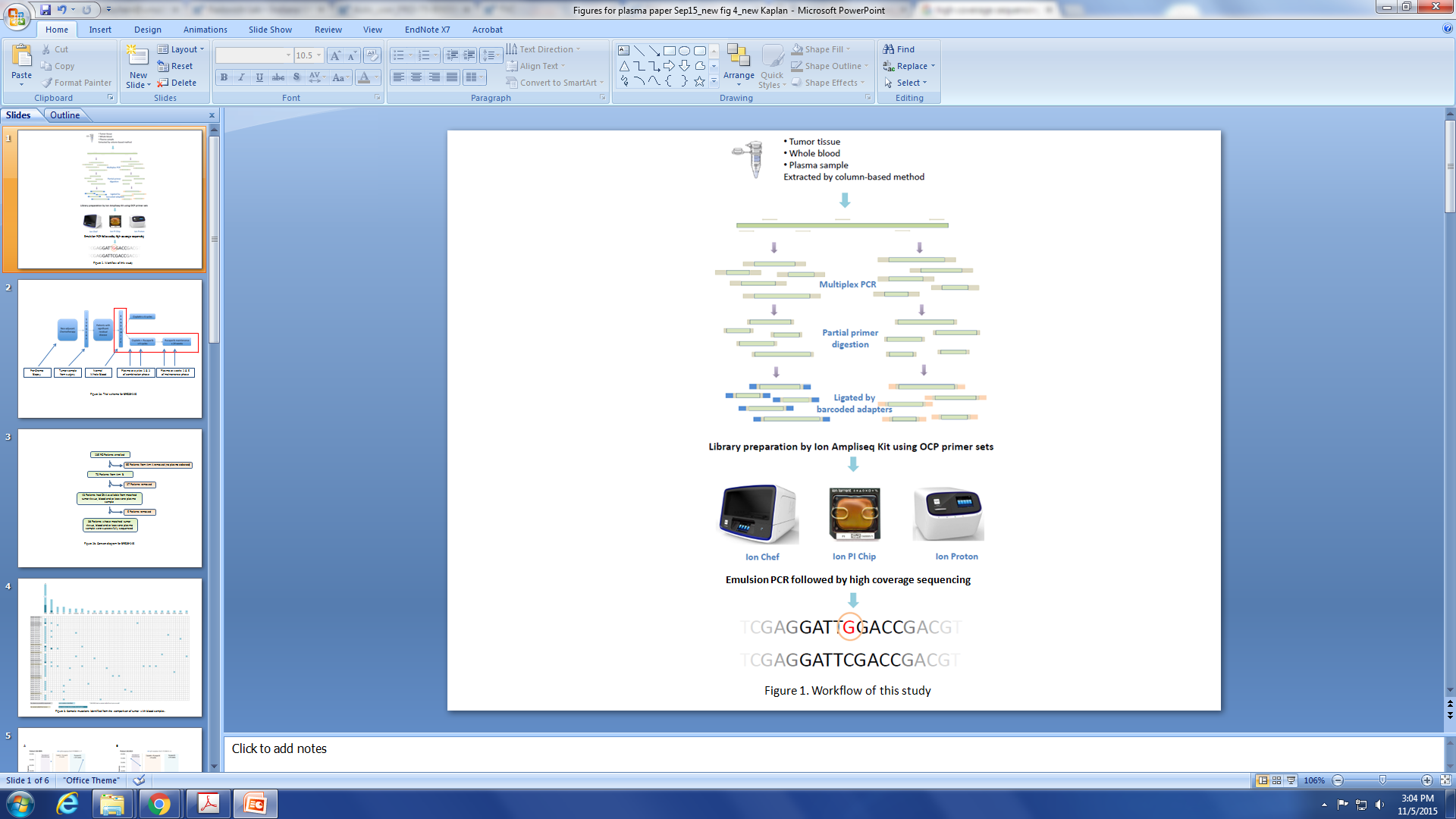
**

**Supplementary Figure 1.  Experimental workflow of mutation identification.**

DNA from tumor tissue, whole blood and plasma samples was extracted by a column-based method.  Isolated DNA was amplified parallelly using the Ion Ampliseq Oncomine Cancer Panel which comprises of 134 cancer-related genes.  Amplicons from different samples of the same patient were further ligated by barcoded adapters so that multiple samples could be analyzed in one sequencing run.  Libraries were then processed by an Ion Chef for preparation for sequencing on an Ion Proton next-generation sequencer using the Ion PI chip.  Mutations were called using the Torrent Suite v4.2.1 and Torrent Variant Caller v4.2.1.0 software, followed by manual inspection.
